# B-Cell Pathology in Juvenile Idiopathic Arthritis

**DOI:** 10.1155/2010/759868

**Published:** 2010-12-02

**Authors:** V. Wiegering, H. J. Girschick, H. Morbach

**Affiliations:** ^1^Pediatric Hematology, Oncology and Neurooncology, Pediatric Stem Cell Transplantation Program, Children's Hospital, University of Würzburg, Josef-Schneider-Str. 2, 97080 Würzburg, Germany; ^2^Pediatric Rheumatology, Immunology and Infectious Diseases, Children's Hospital, University of Würzburg, Josef-Schneider-Str. 2, 97080 Würzburg, Germany; ^3^ Vivantes Children's Hospital, Friedrichshain, Landsberger Allee 49, 10249 Berlin, Germany

## Abstract

Juvenile Idiopathic Arthritis (JIA) is the most common cause of chronic arthritis in childhood and adolescents and encompasses a heterogeneous group of different diseases. Due to the promising results of B-cell depleting therapies in rheumatoid arthritis the role of B-cells in autoimmune diseases has to be discussed in a new context. Additionally, experiments in mouse models have shed new light on the antibody-independent role of B-cells in the development of autoimmune diseases. In this review we will discuss the importance of B-cells in the pathogenesis of JIA appraising the question for an immunological basis of B-cell targeted therapy in JIA.

## 1. Introduction

Juvenile Idiopathic Arthritis (JIA) is a heterogeneous condition gathering together distinct forms of chronic arthritis of unknown etiology that begin before the age of sixteen and persist at least six weeks [1]. The common denominator is a chronic inflammatory process affecting the synovia. According to the ILAR-criteria, JIA is currently divided into seven different subtypes by means of clinical and laboratory parameters [2].

Whereas the systemic onset form of JIA (soJIA) is characterized by an exaggerated inflammatory cascade of the innate immune system without evidence of classical autoimmune features (regarded as “autoinflammation”), autoimmune phenomena (autoreactive T-cells as well as autoantibodies) can be detected readily in the poly- and oligoarticular subgroups [3, 4]. Therefore, impingement of immunological tolerance affecting the adaptive immune system can be hypothesized in both the latter subgroups. A distinctive feature of chronic inflammatory arthritis is the presence of synovial lymphocytic infiltrates that play a role in disease pathogenesis by secretion of proinflammatory cytokines and other soluble mediators. Both T- and B-cells are detected in synovial infiltrates from JIA and Rheumatoid Arthritis (RA) patients. Evidence of autoreactive T-cells as well as autoantibodies reacting with several tissue autoantigens has been provided in both diseases [5, 6]. Beside their well-known function as antibody secreting cells, an antibody-independent role for B-cells in disease pathogenesis has been documented by experimental data as well as the promising results of B-cell depleting therapies in RA [7–9]. Therefore, B-cells might be a promising cellular target for future therapeutic options in JIA as well. In this paper, we will focus on the role of B-cells in the pathogenesis of JIA and discuss possible therapeutic implication of B-cells as targets in JIA.

## 2. Autoantibodies

The role of B-cells in autoimmune and chronic inflammatory diseases has been predominantly viewed from the perspective as precursors of autoantibody producing plasma cells. Autoantibodies might be directly involved in tissue damage; alternatively, the formation of immune complexes might trigger chronic inflammation in a genetically predisposed individual. 

Autoantibodies reacting with different tissue autoantigens can be detected in sera of patients with JIA [10–36]. In parallel to seropositive RA, a distinct group of patients with polyarticular onset JIA are characterized by the presence of rheumatoid factor (RF) [1, 28]. These adolescent JIA patients resemble RA patients in terms of clinical as well as immunological parameters. Besides the presence of RF, antibodies against citrullinated proteins (ACPA) can be detected in these patients. In particular, antibodies against cyclic citrullinated peptide (anti-CCP) as well as against mutated citrullinated vimentin (anti-MCV) have been documented in the RF positive polyarticular subgroups of JIA patients, but not in other subgroups [10–12, 14, 18, 27, 28, 32, 35, 37]. These autoantibodies yielded higher specificity in diagnosing RA and might distinguish a characteristic group of polyarticular JIA patients as well [26, 28]. Anti-CCP antibodies seemed to be associated with a more severe disease progress in RA patients [38]. However, due to the low frequencies of JIA patients displaying ACPAs, these observations have not been replicated for JIA patients. Therefore, testing for ACPAs should not generally be recommended in the diagnostic work-up of childhood arthritis but might be relevant for predicting a severe disease course in a small group of polyarticular onset JIA patients. 

The presence of antinuclear antibodies (ANAs) indicates loss of tolerance against nuclear autoantigens which is a hallmark in the oligoarticular onset subgroup of JIA patients [19, 23, 39]. However, raised titres of ANAs might be present in the polyarticular subgroup and in psoriatic arthritis as well [1]. Although frequently found in JIA patients, the distinct autoantigens of these ANAs are not identified yet. Antibodies against histones and nonhistone chromosomal proteins have been detected in JIA patients [16, 17, 20–22, 24, 26, 29, 33, 34]. However, the antibody profile seemed to be highly individual and did not correlate with disease subtype. At present, there are no autoantibodies against distinct autoantigens known which could completely explain ANA reactivity found in JIA patients' sera. Therefore, ANA testing is still performed by means of indirect immunofluorescence on fixed epidermoid larynx carcinoma cells (Hep2-cells), and efforts to convert ANA testing to ELISA-technique using recombinant or purified native nuclear antigens have brought conflicting results [40, 41]. Nevertheless, ANA testing still has its place in the diagnostic work-up of childhood arthritis. Several reports have documented the association between the presence of ANAs and uveitis in JIA patients [22, 39, 42, 43]. Therefore, testing for ANAs is recommended in the initial work-up of childhood arthritis for risk stratification of uveitis, and the presence of ANAs has been suggested as a risk factor in the recommendation for uveitis screening in JIA patients [42]. However, ANAs are present in healthy individuals as well and might be transiently increased during viral or bacterial infections. Therefore, the validity of ANA testing in diagnosing JIA is limited due to its low disease specificity. Besides their low specificity in diagnosing JIA per se, ANAs might be an indicator for a distinct JIA subgroup [39]. By effort to improve JIA classification, it has been suggested to define homogeneous subgroups by combining patients with psoriatic, oligoarticular, and RF-negative polyarticular arthritis and exploring the value of variable such as age at onset, symmetry of arthritis, and ANA status [44]. ANA-positive patients seem to be relatively homogeneous in terms of early onset of disease (<6 y/o), predominantly female gender, and high risk for iridocyclitis and asymmetric, predominantly oligoarticular JIA [39]. These clinical findings could be supported by gene expression analysis which indicated a B-cell signature in ANA-positive patients with early-onset JIA [45]. Furthermore, the B-cell signature was present in patients with early-onset RF-negative polyarticular JIA as well. In contrast, patients with late onset oligoarticular or RF-negative polyarticular JIA indicated a myeloid gene expression signature. Therefore, age at onset rather than the number of affected joints might hallmark a distinct JIA subgroup which is characterized by a loss of tolerance against nuclear antigens.

Despite the clinical relevance of autoantibody testing in JIA, the molecular and cellular mechanisms which lead to loss of immunological tolerance and production of autoantibodies are not understood in detail, but underline the central role of B-cells in the pathogenesis of JIA [46]. Much work has been done in characterizing the molecular events leading to the generation of autoreactive immunoglobulins. Immunoglobulin diversity is maintained by several molecular processes including the random recombination of V, D, and J gene segments during the development of B-cells in the bone marrow. These random recombinations of gene segments is inevitably linked with generation of autoreactive immunoglobulins [47]. However, tolerance mechanisms are in place in order to avoid the generation of autoreactive B-cells in healthy individuals [48]. Besides apoptosis and anergy, a second round of V(D)J-recombination in autoreactive B-cells of the bone marrow (receptor editing) seems to be a powerful mechanism to exclude potential harmful receptors from the primary B-cell repertoire [49]. A skewed immunoglobulin kappa light chain repertoire has been detected in naïve peripheral blood B-cells of JIA patients by means of single-cell PCR [50]. In particular, the disturbed kappa immunoglobulin light chain repertoire of these B cells might be sequels of two molecular processes: increased events of secondary V(D)J-recombination and a bias for distinct gene segments during the process of recombination. Therefore, one might speculate that B-cell tolerance might be broken by more than one pathogenic mechanism in JIA B cells. Interestingly, increased events of secondary V(D)J-recombination were also suggested by analysing the kappa:lambda ratio in peripheral blood B-cells of JIA patients [51]. V(D)J-recombination has long been considered to be restricted to early B-cell precursors in the bone marrow. However, the observation of B-cells undergoing V(D)J-recombination in secondary lymphoid organs led to the proposal of receptor diversification taking place in mature activated B-cells (receptor revision) [52, 53]. This secondary rearrangement might promote autoimmunity by an uncontrolled creation of autoreactive antibodies. In this regards it was interesting to observe, that mature peripheral blood B-cells of JIA patients might have the potential to perform receptor revision outside the bone marrow [54]. B-cell tolerance checkpoints during early B-cell development were shown to be defective in RA patients [55]. Summarizing the above-mentioned findings in JIA B-cells, one might assume that B-cell tolerance checkpoints might be broken in JIA as well. However, sophisticated analysis on the level of individual B-cells should be undertaken to prove this hypothesis.

## 3. B-Cells As Effectors: Immunoglobulin Secreting and Antigen Presenting Cells

After being activated, B-cells might differentiate into effector cells, which are immunoglobulin producing plasmablasts/plasma cells and memory B-cells. B-cell development and differentiation follow in a tightly regulated, age-dependent way. Disturbed peripheral B-cell homeostasis indicating aberrant activation and differentiation into effector B-cells has been shown in several autoimmune diseases [56, 57]. Flow cytometric analysis of peripheral blood B-cell populations in JIA patients could discover few differences compared with healthy individuals. Expansion of the minor B-cell subset of CD5^+^ B-cells has been observed in oligoarticular and polyarticular JIA [58, 59]. CD5^+^ B-cells have long been regarded as the human counterpart of the murine B-1 B-cells. These B-cells are characterized by the production of antibodies against bacterial wall components, but also constitute a subgroup of innate-like lymphocytes which seem to be enriched for autoreactive immunoglobulins [60]. Whether CD5^+^ B-cells produce disease-related autoantibodies in JIA is not elucidated, yet. However, rather than being an equivalent of B-1 B-cells, CD5+ peripheral blood B-cells might constitute the so-called pre-naïve B-cells [61]. This newly-described population represents a stage of human B-cell maturation that is before the final stage of negative selection of autoantibody expressing B-cells. This population is both phenotypically and functional distinct from both transitional B-cells and naïve B-cells and also expresses CD5 [62–64]. Additionally, the CD24^++^CD38^++^ transitional B-cell subset seems to be expanded in oligoarticular and polyarticular JIA as well [65]. This disturbed homeostasis of the very early peripheral B-cells as well as the skewed kappa light chain repertoire in naïve B-cells suggests defects in B-cell differentiation and possibly a failure of central B-cell tolerance mechanisms in JIA. 

Hypergammaglobulinaemia in active disease, mainly in patients with oligo- and polyarticular-onset JIA, points to B-cell hyperactivity and has been correlated with clinical disease activity [59]. Patients with oligoarticular and polyarticular JIA demonstrated B-cell hyperactivity concomitantly with increased numbers of circulating HLA-DR^+^ T-cells and showed a positive correlation between the proportion of HLA-DR^+^ CD4^+^ T-cells and IgG. These findings point to the importance of the interaction between hyperactive T-cells and B-cells. In contrast, B-cell hyperactivity in patients with soJIA was not associated with increased numbers of HLA-DR^+^ T-cells and underlines again the presumed different immunologic character of soJIA and oligoarticular/polyarticular JIA.

B-cells may possess their pathogenic function at the site of inflammation as activated effector B-cells. Although the presence of B-cells in the inflamed joint has been well documented in JIA, the clinical relevance is still a matter of debate and relatively few data are available on this particular issue [65, 66]. Lymphocytes diffusely infiltrate or form organized aggregates with or without germinal centre like reactions in the inflamed synovium. Those structures resembling germinal centres of secondary lymphatic tissue (“lymphoid neogenesis”) have been implicated in aberrant B-cell activation and differentiation and were thought to be the site of tolerance breakdown and autoantibody production [67–70]. In RA, lymphoid neogenesis rather seems to be associated with the degree of inflammation than the presence or local production of autoantibodies [71, 72]. In JIA the pathogenic role of lymphoid neogenesis remains unclear. Some data suggest a higher frequency in ANA-positive JIA patients [66]. However, synovial fluid plasmablast accumulation and differentiation did not correlate with serum ANA titres in JIA patients [Morbach et al., unpublished]. Interestingly, at the ocular site of inflammation, local accumulation of plasma cells has been observed in JIA-associated uveitis and might have impact on local antibody production [73].

Besides being precursors of antibody-secreting cells, B-cells are important antigen-presenting and cytokine producing cells regulating T-helper cell differentiation. Additionally, B-cells are involved in the regulation of lymphoid tissue structures and neogenesis as well as dendritic cell function [74]. Antigen presentation, expression of costimulatory molecules, and secretion of cytokines seem to be the paramount mechanisms of B-cell pathology in an antibody-independent context. We and others could demonstrate the accumulation of activated memory B-cells in the joints of patients with JIA [65]. These B-cells have up-regulated the costimulatory molecules CD80 and CD86 and could activate T-cells *in vitro* and secrete Th1-polarizing cytokines [Morbach et al., submitted]. Therefore, an antibody-independent role of B-cells in immunopathology should be suggested of relevance in human chronic inflammatory arthritis of childhood.

## 4. B-Cell-Targeting Therapies

In adulthood, the anti-CD20 monoclonal antibody rituximab has been given for refractory RA. The promising results of these B-cell depleting therapies have provided proof of the concept that B-cells play a central role in driving chronic inflammation in autoimmune diseases [8]. However, the use of B-cell depleting therapies has been reported only anecdotally in JIA patients [75]. Summarizing the data on B-cell pathophysiology in JIA, one might assume that B-cell targeting therapies might also be effective in the treatment of JIA patients. However, since B-cell differentiation follows an age-dependent developmental pathway, effects on the adult B-cell system cannot be directly related to the peculiarities of the B-cell system in childhood. Additionally, the time point of B-cell depletion during disease progress might decide between beneficial or harmful effects [76]. Depletion of B-cells with regulatory capacities has been suggested to be responsible for converse effects of B-cell-targeted therapies [77, 78]. Therefore, B-cell-targeting therapies might be a promising therapeutic option in the treatment of refractory JIA. However, depletion of B-cells in the developing B-cell system of childhood and adolescents should be addressed with caution.

## 5. Summary

Although the appearance of autoantibodies is a common pattern in JIA, less is known about the potential role of B-cells in JIA pathogenesis. Overall, the differential gene expression and clinical observation patterns that distinguish early and late onset JIA patients suggest that different pathologic mechanisms may be active depending on the age of disease onset. B-cell pathology seems to play an important role in early-onset JIA.

Plasma cells are known to differentiate and accumulate at the sites of inflammation in JIA patients. However, it remains unknown whether humoral autoimmunity is the driving force of tissue destruction or, on the contrary, might be an epiphenomenon of local inflammation. Besides plasma cells, B-cells might function as antigen presenting cells and provide costimulation and polarizing cytokines to the synovial T-cells, thereby amplifying pathogenic T-cell responses. Therefore, B-cells function as amplifiers of chronic inflammation in the disease progress of JIA and might be a target of future therapies.
